# Infrared Thermal Imaging for Evaluation of Clubfoot After the Ponseti Casting Method—An Exploratory Study

**DOI:** 10.3389/fped.2021.595506

**Published:** 2021-04-20

**Authors:** Balasankar Ganesan, Joanne Yip, Ameersing Luximon, Paul J. Gibbons, Alison Chivers, Suchita Kothe Balasankar, Raymond Kai-Yu Tong, Rifai Chai, Adel Al-Jumaily

**Affiliations:** ^1^Institute of Textiles and Clothing, The Hong Kong Polytechnic University, Kowloon, Hong Kong; ^2^School of Biomedical Engineering, University of Technology Sydney, Sydney, NSW, Australia; ^3^EMEDS Limited, Kowloon, Hong Kong; ^4^Orthopaedic Department, The Children's Hospital at Westmead (Sydney Children's Hospitals Network), Sydney, NSW, Australia; ^5^Physiotherapy Department, Children's Hospital at Westmead, Sydney, NSW, Australia; ^6^Department of Ayurveda, Maharashtra University of Health Sciences, Nashik, India; ^7^Department of Biomedical Engineering, The Chinese University of Hong Kong, Shatin, China; ^8^Department of Telecommunications, Electrical, Robotics and Biomedical Engineering, Swinburne University of Technology, Hawthorn, VIC, Australia

**Keywords:** clubfoot, Ponseti method, infrared thermography, evaluation, thermal imaging, thermography

## Abstract

**Background:** Conservative treatment, Ponseti method, has been considered as a standard method to correct the clubfoot deformity among Orthopedic society. Although the result of conservative methods have been reported with higher success rates than surgical methods, many more problems have been reported due to improper casting, casting pressure or bracing discomfort. Nowadays, infrared thermography (IRT) is widely used as a diagnostic tool to assess musculoskeletal disorders or injuries by detecting temperature abnormalities. Similarly, the foot skin temperature evaluation can be added along with the current subjective evaluation to predict if there is any casting pressure, excessive manipulation, or overcorrections of the foot, and other bracing pressure-related complications.

**Purpose:** The main purpose of this study was to explore the foot skin temperature changes before and after using of manipulation and weekly castings.

**Methods:** This is an explorative study design. Infrared Thermography (IRT), E33 FLIR thermal imaging camera model, was used to collect the thermal images of the clubfoot before and after casting intervention. A total of 120 thermal images (Medial region of the foot–24, Lateral side of the foot–24, Dorsal side of the foot−24, Plantar side of the foot−24, and Heel area of the foot–24) were collected from the selected regions of the clubfoot.

**Results:** The results of univariate statistical analysis showed that significant temperature changes in some regions of the foot after casting, especially, at the 2nd (M = 32.05°C, SD = 0.77, *p* = 0.05), 3rd (M = 31.61, SD = 1.11; 95% CI: 31.27–31.96; *p* = 0.00), and 6th week of evaluation on the lateral side of the foot (M = 31.15°C, SD = 1.59; 95% CI: 30.75–31.54, *p* = 0.000). There was no significant temperature changes throughout the weekly casting in the medial side of the foot. In the heel side of the foot, significant temperature changes were noticed after the third and fourth weeks of casting.

**Conclusion:** This study found that a decreased foot skin temperature on the dorsal and lateral side of the foot at the 6th week of thermography evaluation. The finding of this study suggest that the infrared thermography (IRT) might be useful as an adjunct assessment tool to evaluate the thermophysiological changes, which can be used to predict the complications caused by improper casting, over manipulative or stretching and casting-pressure related complications.

## Introduction

Clubfoot is a common pediatric congenital foot deformity that affects 0.6–1.5 newborn babies in every 1,000 live births, and it mostly occurs in the lower-middle-income countries (1, 2). The surgical methods have been replaced by using non-operative methods in the recent decades (3–7). Among various types of non-operative methods (Ponseti method, Kite method, and the French functional physical therapy method), Ponseti method has been considered as a gold standard method for treating clubfoot. The Ponseti method consists of two phases: (1) Treatment phase (2) Maintenance phase. The treatment phase is involved with gentle manipulation and weekly castings for the period of 6 weeks, and percutaneous Achilles tenotomy (PAT) for correcting the equinus deformity and achieving dorsiflexion of the foot (8–12). The maintenance phase is involved with bracing after the casting phase to prevent the recurrence of the corrected foot. For the first 3 months, the brace needs to be worn for 23 h per day and then the brace needs to be worn only during the night-time upto 5 years (12, 13). Some studies reported that bracing schedule is varied from the original protocol of Ponseti techniques (14, 15). Although non-operative treatment methods showed that less complications and higher success rate than surgical methods in correcting clubfoot deformity, there is still number of complications occur due to improper casting or casting pressure or uncomfortable bracing. For example, Chotigavanichaya et al. study (2016) reported that 5.48% of feet had complications due to loosening of the casting, and 5.48% of feet had skin irritation, cast-associated pressure sore (4.11%), and infection (2.73%) (16). Another study reported that one child had rocker-bottom foot deformity, and another child had blister complication after the casting, and two children had blister complication due to foot abduction orthosis (17). Some patients were suffered by heal sores due to bracing (18). Another study reported that excoriation of the skin was observed in seven feet out of 53 feet (19). A previous study suggested that neurovascular assessment including the checking the margin of the casting needs to be carried out to prevent the swelling, bluish discoloration of the toes after the casting (4), including other complications such as injuries to the lesser saphenous vein peroneal artery (PA), posterior tibial artery (PTA), and incomplete release of Achilles tendon (AT). These kinds of complications might be predicted through assessing the temperature of the affected areas of human body parts. Because, the correlation between diseases and human body temperature is well-established in the last centuries. Due to advanced development of camera technologies, sensors and computing software technologies, infrared thermography (IRT) has been widely used to detect the human diseases such as musculoskeletal disorders or injuries, inflammatory or arthritic conditions, skin problems, vascular diseases, and cancers. The IRT method is a painless, non-invasive, non-radiating exposure device, less expensive and easier to handle as normal camera (20). Initially, the first medical application of IRT was used for the diagnosis of breast cancer in 1956 (21), and then followed by thermal imaging evaluation for arthritic conditions in 1959 (22, 23). Currently, there are number of studies used infrared thermography to screen existing medical conditions or progress of the disease, and evaluation of the effectiveness of treatment methods (20). For instance, thermography evaluation for coccygodynia after manipulative therapy (24), ankle sprains (25), open heart surgeries, reflex sympathetic dystrophy syndrome (RSD), and neurological conditions (26), deep vein thrombosis (27, 28), soccer athletes injuries (29, 30), muscle injuries (31), as a diagnostic evaluation and validation tool for osteopathic management in backpain (32), tendinopathy (33), gastrocnemius-soleus equinus (30), postoperative evaluation for total knee arthroplasty (34), hand-osteoarthritis (35), rheumatoid arthritis (36), juvenile rheumatoid Arthritis (37), knee- osteoarthritis (38), and for detecting the skin and breast cancer, burns, arthritis and skin allergic conditions (39).

The changes of temperature in skin surface topography of human subjects is generally associated with abnormalities of anatomical structures or physiological functions. During the serial castings and manipulations, overcorrection, casting pressure, and other casting related complications might alter the foot skin temperature. Understanding the change of foot skin temperature can help to identify the casting pressure related injuries or excessive manipulation of foot correction related injuries. It might also be useful to avoid extra castings, hospitalisations, and expenses. However, to the best of our knowledge, there are no studies used thermography as a tool to evaluate the thermal changes after casting treatment for clubfoot. The objective of our study was to explore the pattern of temperature changes on the clubfoot before and after each week casting treatment.

## Methods

### Study Design

This study was an explorative study, and ethical clearance was obtained from the Sydney Children's Hospitals Network Human Research Ethics Committee, Sydney, Australia. The Human Research Ethics Committee (HREC) reference number is HREC/16/SCHN/163.

### Participants

In this exploratory study design, four children with clubfoot deformities (two females and two males) were recruited by convenience sampling methods from the Children's Hospital at Westmead Sydney Australia. Among four children, two children had bilateral clubfoot and the remaining two children suffered from unilateral clubfoot. The age range of the participants were 1.5–2 weeks. The inclusion of criteria of this study were: (a) Idiopathic clubfoot, (b) both types of clubfoot (bilateral and unilateral clubfoot), (c) Children ≤ 2 years old with clubfoot (d) both gender, (e) untreated clubfoot, (f) willing to participate in this research study (by parents). The exclusion of criteria of this study were: (a) clubfoot with associated medical conditions, (b) Children ≥ 2 years old with clubfoot, (c) Previously treated clubfoot with any types of intervention.

### Procedures

**Figure 1:**
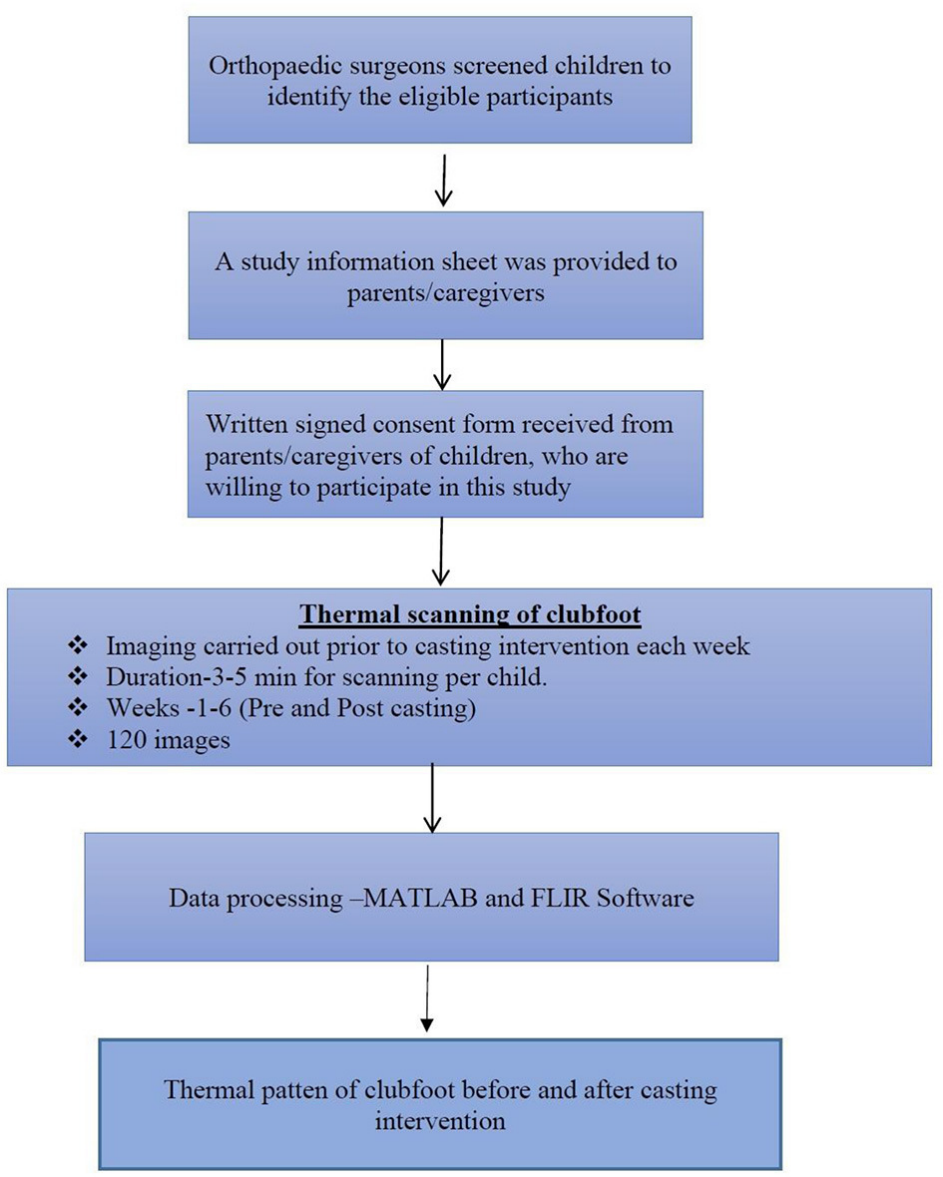
Flow diagram of subjects requirement, data collection, and analysis procedures.

Before starting the experiment, an information sheet of this study was provided to the parent of each participant. Then, each step of the experimental procedures were also explained to each participant of the parents by the investigator. A written informed consent form was obtained from each parent of the participants, those who are willing to participate in this study. The flow chart.1 shows that the methods of subjects requirement, data collection procedures, and data processing of this study (Figure 1). In the treatment phase of Ponseti method, the orthopedic surgeon used manipulative techniques of the Ponseti method and followed by casting was applied on the affected foot.

**Figure 2:**
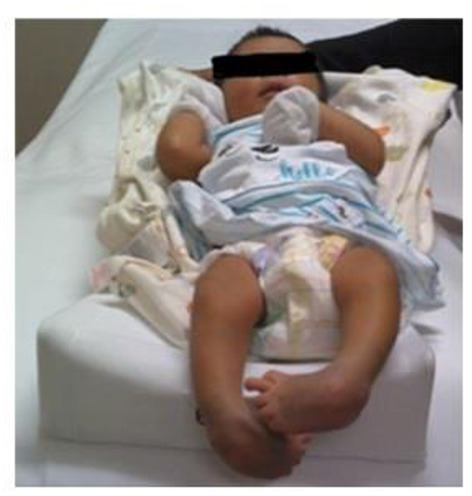
Bilateral clubfoot.

Before performing the thermal imaging experiment, acclimatization period (15 min) was given to each participant based on the thermal imaging experiment protocol of previous studies (25, 40, 41). To normalize the temperature of the feet after removing each cast, the affected feet were cleaned by normal water and followed by all participants were asked to wait for 10 min in the testing room temperature, and 5 min were spent for explaining the testing procedures to the parents of children. In addition, the testing room temperature was constantly maintained at 22°C. As a next step, the participant/child was positioned in a small baby-bathing bed to keep the hip and knee in the slightly flexed position to capture the thermal images. Before positioning the baby, the small bathing bed was positioned at a height of 120 cm on a hospital bed, as shown in Figure 2. In this position, the feet was maintained with the support of small baby-bathing bed without contact.

**Figure 3:**
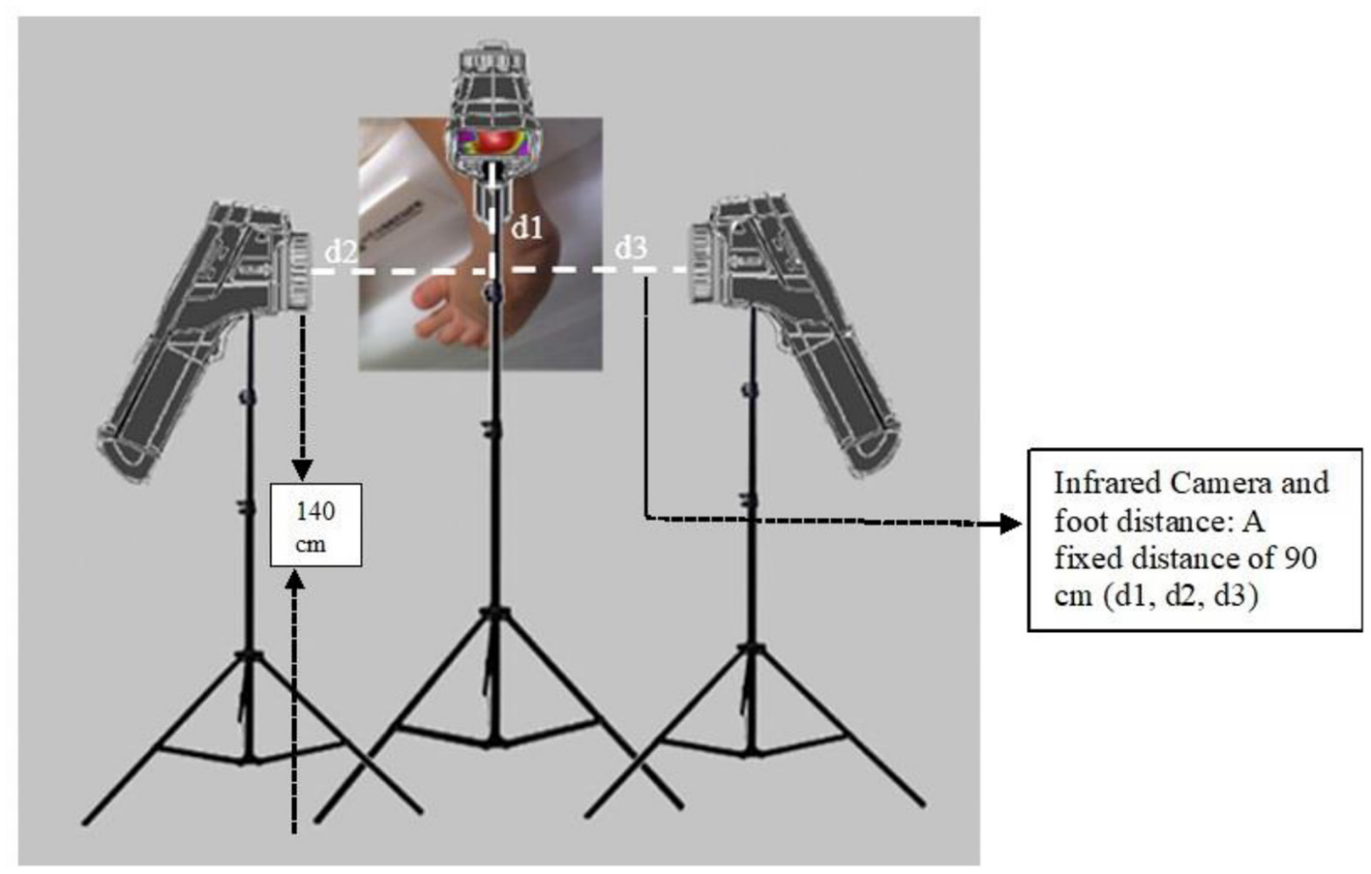
Positioning of IR Camera to capture thermal images (A fixed interval of 90 cm distance (d1, d2, d3) between the camera and foot. The distance between the camera and the floor was 140 cm).

**Figure 4:**
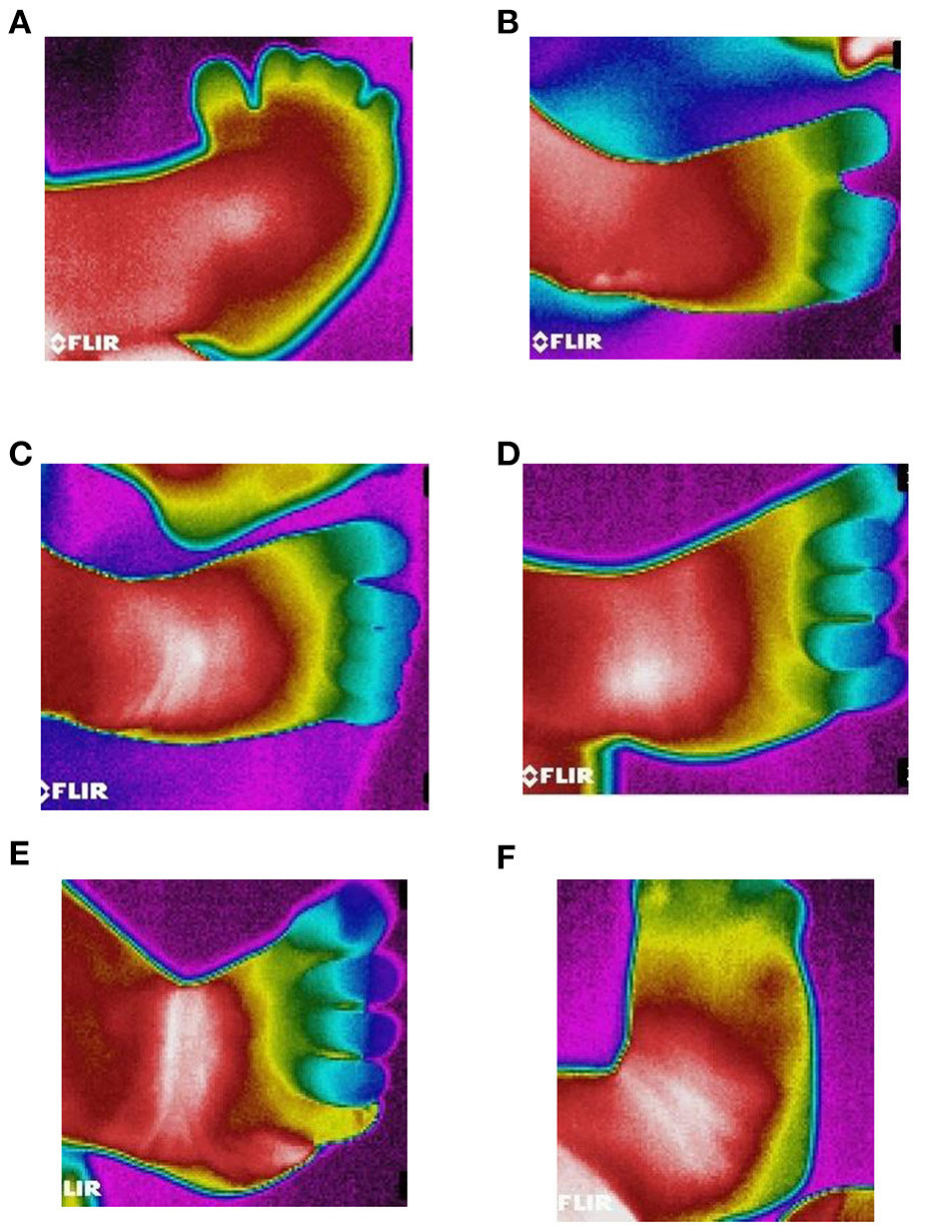
Thermography images of Ponseti serial casting (1–6 weeks). **(A)** Thermal image of clubfoot before casting. **(B)** Thermal image of clubfoot after first casting. **(C)** Thermal image of clubfoot after second casting. **(D)** Thermal image of clubfoot after third casting. **(E)** Thermal image of clubfoot after fourth casting. **(F)** Thermal image of clubfoot after fifth casting (Week 6).

A model-E33 (FLIR Systems) infrared camera (IR) was used to capture the thermal images from the affected foot (Peiport Scientific Limited, FLIR). As shown in Figure 3. IR camera was positioned (d1-front view; d2, d3-side view positions) with the support of tripod to capture the infrared images of the foot. IR camera was positioned directly perpendicular to the selected region of the foot, and a fixed range of 90 cm distance (d1, d2, d3) was used between the IR camera and feet. The distance between camera and floor was 140 cm. The thermal images were collected before the casting intervention to 6th weeks of casting. The thermal sensitivity/noise equivalent temperature difference (NETD) specifications of the IR Camera: <0.07°C @ +30°C (+86°F)/70 mK; measurement uncertainty: ±2°C (±3.6°F) or ±2% of reading, for ambient temperature 10–35°C (+50°F to 95°F); focal plane array sensor size: 160 × 120; minimum focus distance: 25° × 19°/0.2 m (0.66 ft). The thermal images were collected before and after the casting intervention in the following areas of the right-side clubfoot including medial, lateral, dorsal, plantar, and heel side of the foot (Figure 4). The manipulation technique and casting procedures were involved in the medial, lateral, dorsal, plantar, and heel side of the foot. Therefore, we collected thermal images in the region of interested areas of the foot. In the thermal image, blue color represents the cooler area, whereas red color represents hottest areas (42). For collecting the thermal image from the heel side of the foot, parents were asked to hold the baby close to their chest. It took about 3–5 min to position the participant and collect the thermal images.

### Data Processing and Statistical Analysis

**Figure 5:**
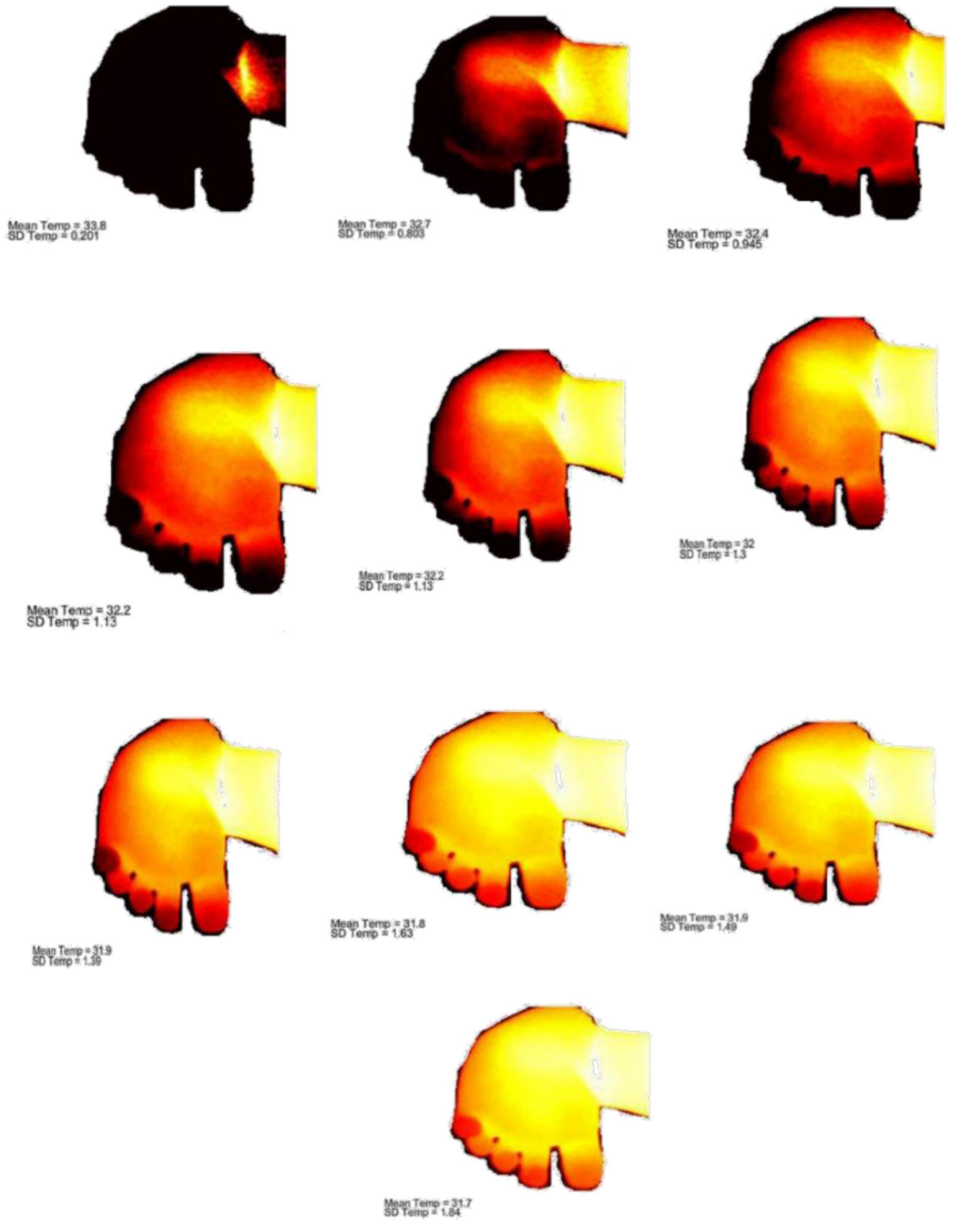
Cut off temperature scores (maximum and minimum skin temperature) of the selected region of the foot.

The temperature data (CSV file) were collected from the thermal images using FLIR Tools Software. Simultaneously, the Microsoft Paint- software was utilized to clean and hide the unwanted areas of thermal images. Then, the cleaned images, uncleaned images, and CSV temperature data files were computed in the MATLAB (MATLAB 2017a-Mathworks Inc, MA, USA) to obtain the mean temperature of the selected areas in the foot. Ten cut off temperature scores were obtained from each region of interested area of the foot, and the yellow color region will have highest temperature in the processed infrared image as shown in the Figure 5. The mean temperature of the 10 cut off temperature in the selected region of the foot were taken to the statistical analysis. As a final step of data analysis, SPSS software (Statistical Package for the Social Sciences) was used for statistical analysis. Univariate analysis is used and *p*-value of <0.05 was set for statistical significance. The 10 cut off temperature score (mean temperature) and weekly casting were selected as fixed factors, and temperature data was used as dependent variable. In *post-hoc* analysis, least significance difference (LSD) tests at a 95% confidence interval was performed to find the relationship between the weekly casting intervention and thermal changes in the foot.

## Results

A total of 120 thermal clubfoot images (Medial side of the foot–24, Lateral side of the foot–24, Dorsal side of the foot−24, Plantar side of the foot−24, and Heel area of the foot–24) were included in the study, and those clubfoot thermal images were obtained in the medial, lateral, dorsal, and plantar heel areas of the clubfoot at the pre-casting stage and prior to the 2–6th weeks of casting intervention. Amongst four children, age at the time of intervention ranged from range 1.5–2.0 weeks, and the mean age of participants were 1.75 weeks. In this study, 50% of participants were female, and 50% had bilateral clubfoot. Also, we noticed that fifty percentages were bilateral clubfoot in both genders. Table 1 shows the result of the skin temperature changes in the dorsal, plantar, heel, medial and lateral side of the clubfoot before and after weekly casting intervention.

**Table 1:** Thermal changes (mean ± standard deviation) in the clubfoot before and after casting intervention.

**Thermal changes in the clubfoot (Week/Casting 1: Pre-Intervention; Week/Casting 2–6: Post Intervention)**						
**Region of interest (ROI) in the foot**	**Week/** **casting**	**Mean (°C)**	**Std**	**95% confidence interval lower bound**	**95% confidence interval upper bound**	***P*****-value**
Dorsal side	1	32.26	0.936	31.85	32.678	-
	2	31.88	0.862	31.4	32.301	0.153
	3	31.78	1.13	31.36	32.195	0.067
	4	32.21	1.21	31.80	32.632	0.862
	5	31.71	2.08	31.238	32.195	0.055
	6	30.67	1.67	30.197	31.153	0.000
Heel side	1	31.438	1.64	31.142	31.734	-
	2	30.899	0.82	30.603	31.195	0.01
	3	31.787	1.17	31.492	32.083	0.01
	4	30.727	0.81	30.431	31.023	0.001
	5	32.932	1.09	32.591	33.274	0.00
	6	31.595	1.13	31.254	31.937	0.49
Lateral side	1	32.39	1.01	32.054	32.74	-
	2	32.054	0.77	31.709	32.39	0.057
	3	31.6195	1.11	31.274	31.96	0.000
	4	32.1995	1.07	31.854	32.54	0.269
	5	32.6650	1.04	32.267	33.06	0.172
	6	31.1500	1.59	30.752	31.54	0.000
Medial side	1	31.7	1.26	31.236	32.166	-
	2	31.08	1.11	30.624	31.553	0.59
	3	31.17	0.943	30.707	31.637	0.10
	4	31.82	0.71	31.362	32.291	0.69
	5	31.27	2.93	30.734	31.808	0.21
	6	31.90	1.27	31.365	32.439	0.56
Plantar side	1	31.36	1.27	31.00	31.73	-
	2	31.12	1.22	30.75	31.48	0.20
	3	31.08	0.86	30.71	31.44	0.20
	4	31.98	0.84	31.61	32.34	0.02
	5	32.59	1.62	32.17	33.01	0.00
	6	31.28	1.12	30.86	31.70	0.68

### Medial and Lateral Side of the Foot

**Figure 6:**
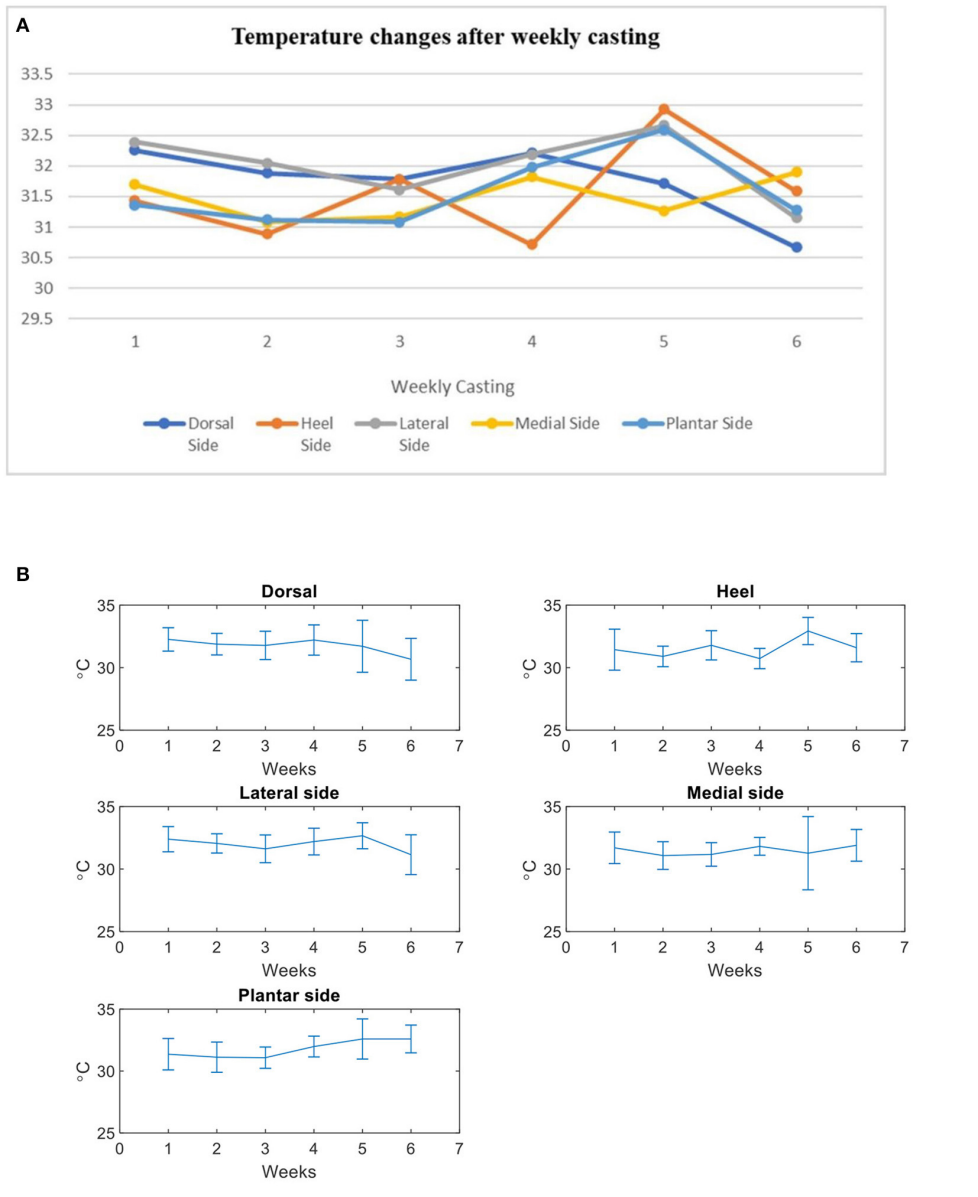
Thermal changes before and after weekly casting. **(A)** Temperature changes after casting (All subjects). **(B)** Mean and standard deviation (error bars) of temprature changes after casting (Pre casting- Post casting intervention: 1–5 casts).

In the medial side of the foot, the results of LSD test showed that there was no significant difference in the mean temperature at the post-intervention (6th week of evaluation) (M = 31.90, SD = 1.27, 95% CI: 31.36–32.43, *p* = 0.56), as well as in the 2nd (*p* = 0.044) to 5th (*p* = 0.25) weeks of casting intervention. Figures 6A,B shows the thermal changes between each week casting in the region of interested areas (ROI) of the foot.

In contrast to the dorsal side of the foot, a significant mean-temperature differences were found at the 2nd (M = 32.05°C, SD = 0.77, 95% CI: 31.70–32.39, *p* = 0.05), 3rd (M = 31.61, SD = 1.11, 95% CI: 31.27–31.96, *p* = 0.00), and 6th week of evaluation on the lateral side of the foot (M = 31.15°C, SD = 1.59, 95% CI: 30.75–31.54, *p* = 0.000). Of these 6 weeks of thermal evaluation on the lateral side of foot, we found that there was an increased mean temperature after removal of the 4th week-casting (5th week of evaluation; M = 32.66°C, SD = 1.04, 95% CI: 32.26–33.06, *p* = 0.17). However, the mean temperature was not statistically significant at the 5th week of evaluation.

#### Heel Side of the Foot

The results of the thermographic evaluation study on the heel side of foot showed that there was no significant difference in the mean temperature on the heel side of clubfoot (6th week of casting, M = 31.59°, SD = 1.13, 95% CI: 31.2–31.9, *p* = 0.49). However, we found that there was a significant difference in the mean temperature at the 2nd (*p* = 0.01), 4th (*p* = 0.00), and 5th (*p* = 0.00) weeks of casting intervention as shown in Table 1. According to the results of *post-hoc* analysis, the mean temperature was statistically significant at the 2nd week (LSD test; M = 30.899°, SD = 0.82, 95% CI: 30.60–31.195, *p* = 0.01), 4th week (LSD test; M = 30.72, SD = 0.81, 95% CI: 30.43–31.02, *p* = 0.001), and 5th week (LSD test; M = 32.93, SD: 1.09, CI: 32.59–33.27, *p* = 0.00). However, there was no statistic difference between pre and post casting intervention in the *post-hoc* tests (LSD test; M = 31.59°, SD = 1.13, 95% CI: 31.2–31.9, *p* = 0.49).

## Discussion

In this study, we examined the clubfoot skin temperature before and after clubfoot casting intervention. Understanding of thermo-physiological changes (casting, tenotomy, and bracing techniques) after each stage of clubfoot casting intervention can be helpful in preventing complications caused by improper castings and bracings. Preventing casting and bracing related complications might be useful to increase the success rate of conservative treatment methods of clubfoot. Although casting related complications are less likely to occur in the Ponseti methods than surgical corrections to correct the clubfoot deformity, the wound infections, pain, tenderness, and skin irritation are unavoidable in some cases. Several studies reported that the occurrence of minor complications are common in the corrective casting phase of clubfoot treatment, such as skin lesions (11), slippage of casts (16), ulceration due to tight casts and plaster sores (5), but there is no clear understanding of what weeks these casting complications occur. Other complications in the clubfoot correction, such as relapses (43, 44), tenderness, pain, mild infections (45), post tenotomy infection (46), and pseudoaneurysm (47) have been reported in the previous studies. Furthermore, excessive manipulation when using casting procedures with children under anesthesia or sedation is likely to cause complications such as skin irritation, bruising and ulcers (15). Therefore, early identification of skin foot temperature changes would be useful to avoid the serious complications, and this imaging method can be used as a diagnostic tool to predict the complications during the treatment and maintenance phase of Ponseti method. Although infrared thermography does not reveal the anatomical structural abnormalities, but it helps to find the physiological changes (skin temperature) due to structural abnormalities, injuries, pain, and tenderness. In our study, we found that the functional or physiological changes of the foot after each week of casting techniques. The first casting was aimed to correct the cavus by aligning the forefoot with hindfoot (48), therefore, forefoot was supinated with applying gentle pressure on the first metatarsal area of the foot. After implementing the manipulation and the 1st week of casting intervention, we found that there was a significant temperature change in the heel and lateral side of the foot. In the following week, the second cast was applied to correct the cavus deformity (13). Unlike the 1st week of casting, there was no significant temperature changes in any selected regions of the foot after the 2nd week of casting. Then, third and fourth castings were done by our investigator to correct the hindfoot varus, equinus, and forefoot adduction. It was performed by using the following methods: forefoot was abducted in supinated position as a first step and then, simultaneously counter pressure was given on the head of the talus. Generally, tenotomy will be recommended before applying fifth cast if dorsiflexion is limited (49). After 3rd and 4th weeks of casting, significant temperature changes were noticed on the heel side of the foot or hindfoot. In view of dorsal side of the foot, it was found that there was a significant temperature change after fourth and fifth casts. However, no significant temperature changes were observed throughout the casting stages (1–5 casts) in the medial of the foot. After removal of casts, thermographic evaluation can be used as a diagnostic tool to identify the skin irritation, swelling, or other complications due to casting or excessive manipulation. Although several thermographic studies have been proposed to assess various musculoskeletal injuries (39), to the best of our knowledge, this is the only and first study explored the thermal changes (skin temperature) after applying manipulation and corrective serial casting techniques in clubfoot. In addition, we did not observe any musculoskeletal injuries during or after the corrective serial casting techniques. However, overall, this study found that decreased foot skin temperature in the dorsal and lateral side of the foot at the 6th week of clubfoot-thermal evaluation. Previous studies found that the dorsal side of mean foot skin temperature among healthy children was 30.7°C and planter side was 28.7°C (50). In our study, we found that the mean dorsal side foot skin temperature was 32.2°C, which is higher than the normal mean foot skin temperature. After 5 weeks of casting treatment for clubfoot, the mean dorsal side foot skin temperature dropped to 30.6°C as the normal mean dorsal foot skin temperature. Similarly, the mean planter side foot skin temperature in our findings was 31.36°C, which is higher than normal mean foot skin temperature (28.7°C). However, our findings showed that there were no significant changes in plantar side foot skin temperature (31.28°C) after 5 weeks of casting treatment.

This study has several strengths and limitations. The strength of the exploratory study is the consistency of treatment for correcting the clubfoot deformity. Obtaining thermal images or 3D scanning from a newborn baby is a difficult task, due to moving the feet frequently by babies. In addition, it is difficult to put a newborn baby in a weight-bearing position. Therefore, we have adapted a small bed, which helped to keep the knee in a slightly flexed position, and it helped to capture the thermal images in the selected regions of the foot, including planter side of the foot. However, our study has some limitations. The first limitation of this exploratory study is that small sample size. In our study, thermal images were collected only from the right side of the clubfoot, so this study did not compare the thermal changes between normal foot and clubfoot, and the contralateral foot. Another limitation of this study is that the thermal changes were not assessed during the maintenance phase of the Ponseti method (bracing techniques).

## Conclusion

In conclusion, our study found that after casting treatment of clubfoot, a decreased foot skin temperature was noticed on the dorsal and lateral side of the foot. However, there is still much to learn about the weekly casting intervention, bracing and clubfoot deformity. In this current study, we used infrared thermography (IRT) as an assessment tool to evaluate the thermophysiological changes in the clubfoot. Based on the findings, this study suggests that adding the foot skin temperature evaluation, in addition to the current subjective evaluation methods, may be helpful in detecting abnormalities, and avoiding complications of improper casting, excessive manipulation, and other bracing pressure-related infections. Furthermore, more research with large sample size is needed to understand more about thermophysiological changes in the clubfoot treatment.

## Data Availability Statement

The raw data supporting the conclusions of this article will be made available by the authors, without undue reservation.

## Ethics Statement

This study was an explorative study, and ethical clearance was obtained from the Sydney Children's Hospitals Network Human Research Ethics Committee, Sydney, Australia. The Human Research Ethics Committee (HREC) reference number is HREC/16/SCHN/163. Written informed consent to participate in this study was provided by the participants' legal guardian/next of kin.

## Author Contributions

BG, AL, JY, and AA-J contributed to the conception and design of the study. BG, AA-J, PG, and AC contributed to apply and obtain ethical procedures of this study. BG, SB, PG, and AC collected the data. BG, JY, AL, AA-J, SB, and RC helped to analyse and interpret the data. RT helped to interpret the data, edit, and revised the manuscript. BG drafted the main manuscript. All authors contributed to development of the final submitted manuscript.

## Conflict of Interest

AL was employed by company EMEDS Limited. The remaining authors declare that the research was conducted in the absence of any commercial or financial relationships that could be construed as a potential conflict of interest.
